# *Klebsiella pneumoniae* prevents spore germination and hyphal development of *Aspergillus* species

**DOI:** 10.1038/s41598-018-36524-8

**Published:** 2019-01-18

**Authors:** M. F. Nogueira, L. Pereira, S. Jenull, K. Kuchler, T. Lion

**Affiliations:** 1 CCRI - Children’s Cancer Research Institute, Vienna, Austria; 2 Labdia - Labordiagnostik GmbH, Vienna, Austria; 3 MFPL - Department of Medical Biochemistry, Medical University of Vienna, Max F. Perutz Laboratories, Campus Vienna Biocenter, Vienna, Austria; 4 Department of Pediatrics, Medical University of Vienna, Vienna, Austria

## Abstract

Different bacteria and fungi live as commensal organisms as part of the human microbiota, but shifts to a pathogenic state potentially leading to septic infections commonly occur in immunocompromised individuals. Several studies have reported synergistic or antagonistic interactions between individual bacteria and fungi which might be of clinical relevance. Here, we present first evidence for the interaction between *Klebsiella pneumoniae* and several *Aspergillus* species including *A. fumigatus, A. terreus, A. niger* and *A. flavus* which cohabit in the lungs and the intestines. Microbiological and molecular methods were employed to investigate the interaction *in vitro*, and the results indicate that *Klebsiella pneumoniae* is able to prevent *Aspergillus* spp. spore germination and hyphal development. The inhibitory effect is reversible, as demonstrated by growth recovery of *Aspergillus* spp. upon inhibition or elimination of the bacteria, and is apparently dependent on the physical interaction with metabolically active bacteria. Molecular analysis of *Klebsiella*-*Aspergillus* interaction has shown upregulation of *Aspergillus* cell wall-related genes and downregulation of hyphae-related genes, suggesting that *Klebsiella* induces cell wall stress response mechanisms and suppresses filamentous growth. Characterization of polymicrobial interactions may provide the basis for improved clinical management of mixed infections by setting the stage for appropriate diagnostics and ultimately for optimized treatment strategies.

## Introduction

Microbial interactions are part of the highly complex human microbiome. Mapping of the human microbiome has shown a wide diversity of bacteria and fungi occupying specific niches^1–4^. The interplay between various microorganisms and their interactions with the host and the immune system may display beneficial or harmful effects. Importantly, changes induced by alterations including, for example, underlying diseases, the use of antibiotics, anti-cancer chemotherapy, or dietary changes have an enormous impact on microbial populations^1,3,5–11^. More recently, polymicrobial infections and bacterial-fungal interactions (BFI) have attracted greater attention. Several such interactions involving particularly *Candida (C.) albicans* have been reported^12–20^. The well-studied interaction between *C. albicans* and *Pseudomonas (P.) aeruginosa* has shown that synergistic and antagonistic effects can occur simultaneously, and the net results of the interactions can vary depending on external influences and the dominant intermicrobial dynamics^12–14,21–24^. A number of other bacteria were shown to interact with *C. albicans*, including *Streptococcus* spp., *Lactobacillus* spp., *Staphylococcus (S.) aureus*, *Enterococus faecalis*, and *Escherichia coli*^12,15–17,19,20,25–27^. *P. aeruginosa* has the capacity to inhibit the growth of various fungi, such as *Aspergillus (A.) fumigatus* and *Cryptococcus* spp^28–34^. These interactions occur through the production of quorum sensing molecules and virulence factors by *P. aeruginosa* including e.g. phenazines, decanol and 3-oxo-C12-homoserine lactone (3OC12HSL), which affect biofilm formation, inhibit yeast (*Cryptococcus* spp.) and hyphal development (*C. albicans*, *A. fumigatus*) through the generation of highly toxic reactive oxygen species (ROS)^12–14,21–24,28–34^. Adherence of *P. aeruginosa* and *S. aureus* to the hyphal form of *C. albicans* is thirty times higher compared to the yeast form^16^. The indicated interactions between bacteria and fungi occur when these pathogens share the same niches. Moreover, co-localization of *A. fumigatus* and *P. aeruginosa* in the lungs of patients with cystic fibrosis was associated with poorer outcomes when compared to single infections with these pathogens^35,36^. The same effect was observed in various studies reporting on the interaction of *C. albicans* with *P. aeruginosa*, which resulted in elevated mortality rates^14,21,37^, in line with other reports on polymicrobial infections^16^. Bacteria and fungi often live in highly organized structures termed biofilms rather than in planktonic state. Biofilms pose a higher risk for the development of serious infections because they often display greater resistance to antimicrobial treatment and to control by the immune system^38–43^. The biofilm structure protects against exogenous stresses including drug treatment, and provides a microenvironment facilitating nutrition and quorum sensing communication. Such features improve the fitness and resilience of biofilm structures providing a survival advantage in the host^43–51^. In our studies, we aimed to investigate the interactions between select opportunistic fungal pathogens including different *Aspergillus* species and the clinically important bacterium *Klebsiella pneumoniae*. Both species cohabit in various regions of the body including particularly the gut and the lungs^1,2,52,53^. Development of *Aspergillus* species encompasses the formation of spores or conidia and hyphal filaments. Under favorable environmental conditions, spores germinate into long hyphae which are responsible for tissue invasion and escape from the immune system^54–57^. *Aspergillus* species have the capacity to form biofilms in which hyphae play a leading role. The architecture of the lungs in conjunction with hyphal development of *Aspergilli* makes it difficult for the immune system to clear the fungal pathogens^57–60^. Next to *C. albicans, A. fumigatus* is the most prevalent cause of fungal infection in immunocompromised patients^56,61,62^, and is responsible for approximately 90% of invasive aspergilloses, based on clinical reports^63,64^. More recently, however, shifts from *A. fumigatus* to non-fumigatus *Aspergillus* species have been observed, involving particularly *A. terreus*, *A. niger* and *A. flavus*^65–69^. On the bacterial side, *K. pneumoniae* is an emerging pathogen displaying resistance to antibiotic treatment which has been associated with several nosocomial outbreaks^53,70–83^. The antimicrobial resistance and virulence of numerous clinical strains of *K. pneumoniae* have been associated with the presence of plasmids carrying resistance genes, the hypermucoviscosity phenotype, capsular polysaccharides and the capacity to form biofilms^75,84–90^. Bacteria and fungi are constantly exposed to stress conferred particularly by the host microenvironment and other pathogens. The cell wall is the first point of contact between microorganisms and the host or other pathogens. The cell wall plays an important role in mediating interactions with the external environment relevant for nutrient diffusion and molecule-based signalling. Simultaneously, it protects the cells from oxidative or osmotic stresses, and modulates the response to antimicrobial drugs^91–95^. Fungi are capable of adapting their cell walls in response to stress by activating multiple mechanisms directed towards repair or compensation for cell wall damage. In response to stress, *C. albicans* was shown to activate the MAPK and Ca^2+^/Calcineurin pathways, leading to upregulation of genes involved in the cell wall assembly, and *Aspergillus* species respond to stress in a similar manner^96–104^. It is important to point out that fungal spores and hyphae display different structures and compositions of the cell wall which can induce differential immune responses by the host^105,106^.

In the present study, we sought to investigate the interactions between four *Aspergillus* species including *A. fumigatus*, *A. terreus*, *A. niger* and *A. flavus*, and different strains of *K. pneumoniae* with low or high capacity of biofilm formation. Our studies provide new insights into the biological behavior of *Aspergillus* and *K. pneumoniae* in co-culture, by unravelling the type of interaction and response to stress. Our observations highlight the importance of identifying the presence of polymicrobial infections and potential interactions between the pathogens with regard to optimized diagnostic approaches and appropriate antimicrobial treatment.

## Results

### *K. pneumoniae* inhibits spore germination and hyphal development of *Aspergillus* species

**Figure 1:**
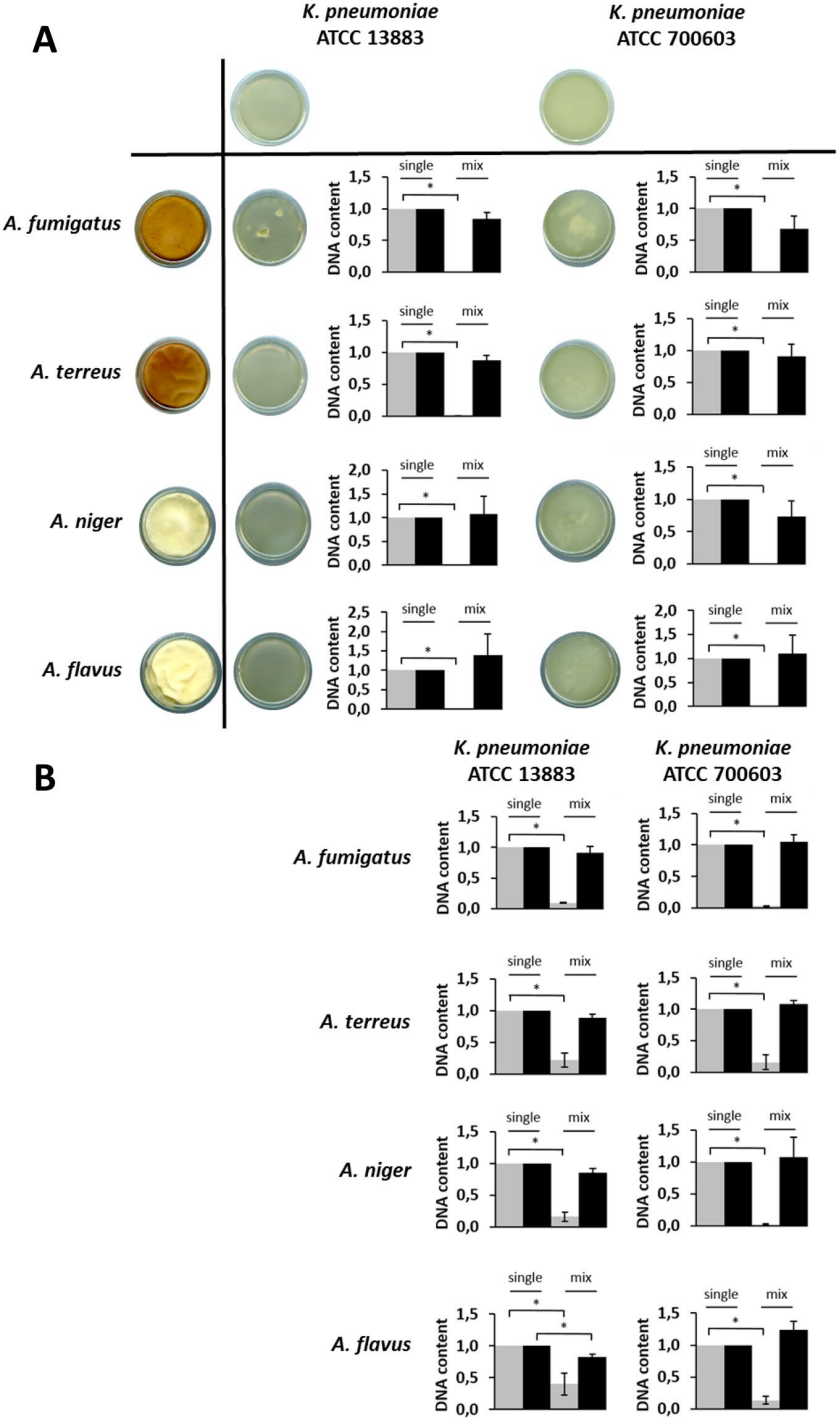
Inhibition of *Aspergillus* spore germination and hyphal development by *K. pneumoniae* strains. (**A**) Fungi and bacteria co-cultured since time zero. Scanner photos of the bottom of culture dishes taken after 48 h of cultivation are shown. (**B**) Fungal spores germinated for up to 12 h followed by addition of bacteria for a total of 24 h. The DNA content was quantified by qPCR at 24 h using species-specific primers for the conserved rRNA sequences 28S and 16S for fungi and bacteria, respectively. The grey bars in the plots correspond to fungi and the black bars to bacteria. The DNA content of single cultures was set to one, and the DNA content of each species in the co-culture was normalized to the corresponding single culture. Data are presented as mean + SE of three independent experiments.

**Figure 2:**
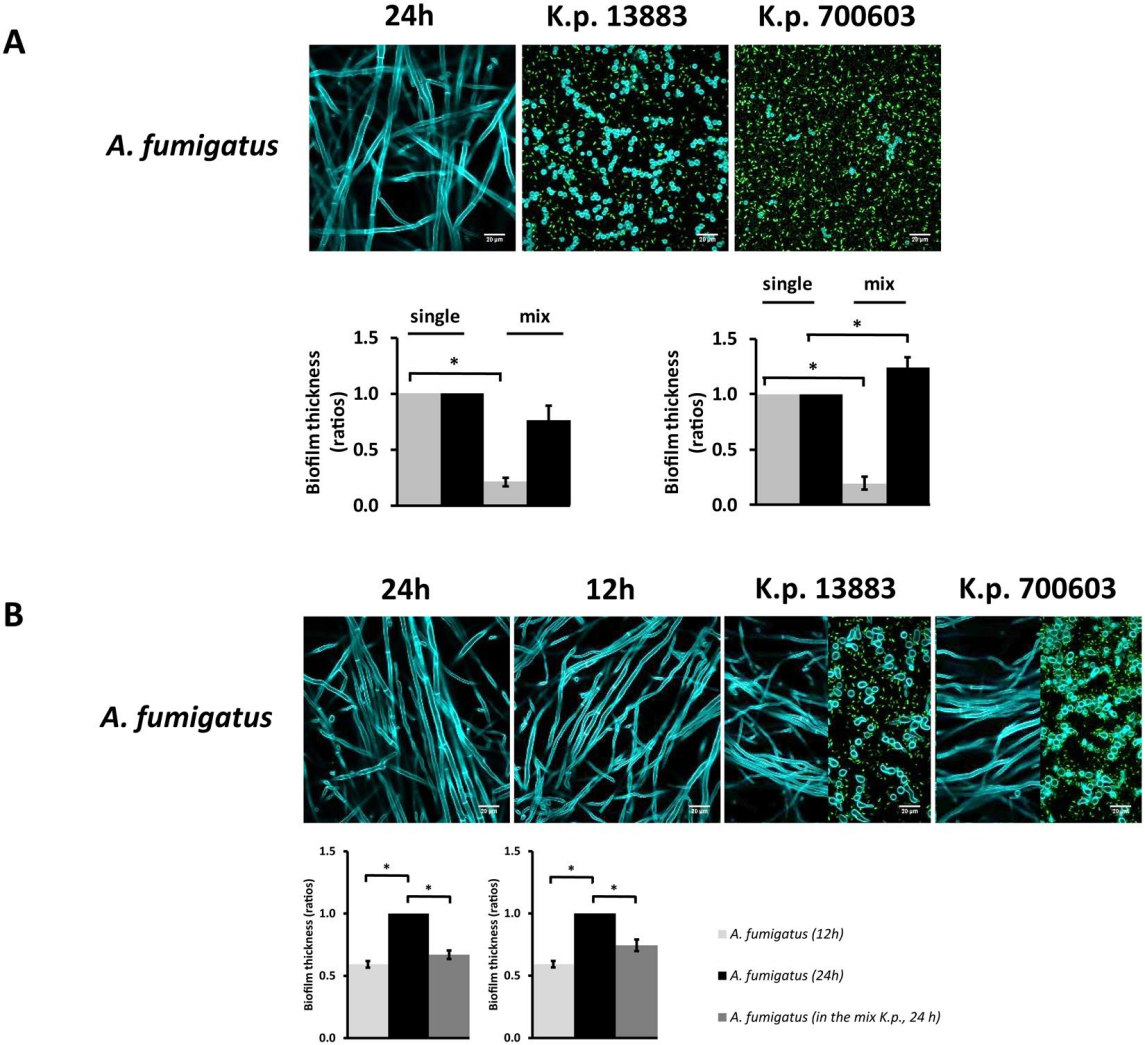
Biofilm thickness of *A. fumigatus* vs *K. pneumoniae* analyzed by confocal microscopy. The fluorescent photos represent *A. fumigatus* growing alone or in co-culture with different strains of *K. pneumoniae*. Differential staining of the microorganisms upon growth in culture and measurement of biofilm thickness was performed as indicated in the Methods section. (**A**) Fungi and bacteria co-cultured since time zero. Grey bars correspond to fungi and black bars correspond to bacteria. (**B**) Fungal spores germinated for up to 12 h followed by addition of bacteria for a total of 24 h. The confocal microscopy images of *A. fumigatus* and *K. pneumoniae* co-cultures (K.p.13883, K.p.700603) show the levels of hyphae (left) and spores (right), respectively. The left plots indicate co-cultures of *A. fumigatus* with *K. pneumoniae* ATCC 13883 and the right plots indicate co-cultures of *A. fumigatus* with *K. pneumoniae* ATCC 700603. Biofilm thickness of the single cultures was set to one and the biofilm thickness of each species in the co-culture was normalized to the corresponding single culture. Data are presented as mean + SE of three independent experiments.

The *in vitro* interaction between *K. pneumoniae* and several *Aspergillus* species, including *A. fumigatus, A. terreus, A. niger* and *A. flavus* was characterized. *K. pneumoniae* and *Aspergillus* were grown alone and in co-culture for 24 and 48 h, as outlined in the Methods section. The level of inhibition of fungal spore germination was assessed by qPCR at 24 h (Fig. 1) and imaged by confocal microscopy (Fig. 2). The suppression of *Aspergillus* growth by *K. pneumoniae* strains is shown in Fig. 1. Our data have shown that, upon contact with *K. pneumoniae*, *Aspergillus* spores are not developing into hyphae (Fig. 1A). This is reflected by the unchanged DNA content of *Aspergillus* in the co-culture which corresponds to the initial fungal cell loads. In addition, this inhibition is independent of the biofilm forming capacity of *K. pneumoniae* strains, since the same effect was observed when using low-biofilm (ATCC 13883) and high-biofilm (ATCC 700603) forming strains. As shown in Fig. 1A, the inhibitory effect was observed for all *Aspergillus* species tested, indicating that this effect is *Aspergillus* species-independent.

Due to the functional importance of hyphae for tissue invasion and escape from the immune system, we have addressed the effect of *K. pneumoniae* on hyphal development of the indicated *Aspergillus* species. We sought to understand whether *K. pneumoniae* also has an effect on hyphal development and if the inhibitory effect still occurs at the hyphal stage. The results have shown that, in the presence of *K. pneumoniae*, *Aspergillus* species were not able to progress with hyphal development by demonstrating significantly decreased DNA contents compared to *Aspergillus* in single cultures (Fig. 1B), revealing that the inhibitory effect of *K. pneumoniae* on *Aspergillus* species is independent of the fungal growth stage.

### Biofilm formation of *Aspergillus* species is affected by the presence of *K. pneumoniae*

Both *K. pneumoniae* and *Aspergillus* species used in this study are capable of forming biofilms. Based on the observation that *K. pneumoniae* strains are able to suppress *Aspergillus* growth and hyphenation, we sought to investigate whether low- and high-biofilm forming strains of *K. pneumoniae* might also affect the biofilm formation of *Aspergillus*. A confocal microscopy approach was used to measure the biofilm thickness of bacteria and fungi growing alone and in co-culture. The results have shown that, upon contact with *K. pneumoniae* strains, the biofilm thickness of *A. fumigatus* was significantly decreased compared to *A. fumigatus* growing alone. These observations are supported by the confocal images showing that *A. fumigatus* in co-culture remained in the form of spores, independently of the *K. pneumoniae* strain tested (Fig. 2A). In addition, the high-biofilm forming strain of *K. pneumoniae* showed thicker biofilms when grown in the presence of *A. fumigatus* (Fig. 2A, right plot). We further investigated whether *K. pneumoniae* strains could also influence the biofilm formation of pre-formed *A. fumigatus* biofilms. To address this question, *A. fumigatus* spores were pre-germinated for 12 h, followed by addition of *K. pneumoniae* strains. Bacteria and fungi were grown alone for comparison with the co-cultures. *A. fumigatus* was grown alone for 12 and 24 h. The plots in Fig. 2B show that there was a significant increase in biofilm thickness between 12 and 24 h, indicative of normal fungal growth. Once bacteria were added to the culture at 12 h, we observed that the biofilm thickness of *A. fumigatus* was significantly decreased in the presence of either bacterial strain. The confocal images showed normal hyphal development at 12 and 24 h. Interestingly, once *A. fumigatus* was growing in co-culture with the *K. pneumoniae* strains, both morphologies of *A. fumigatus* were present, spores and hyphae (Fig. 2B). The images represent different slices of the Z-stacks indicating a level-dependent, predominant presence of hyphae (Fig. 2B, left-half image) or spores (Fig. 2B, right-half image). The density of bacterial cells was greater (green dots in the image) in areas revealing mainly fungal spores, in line with the observation that *K. pneumoniae* strains inhibit both spore germination and hyphal development.

### Inhibition of *Aspergillus* growth by *K. pneumoniae* is dependent on direct contact

**Figure 3:**
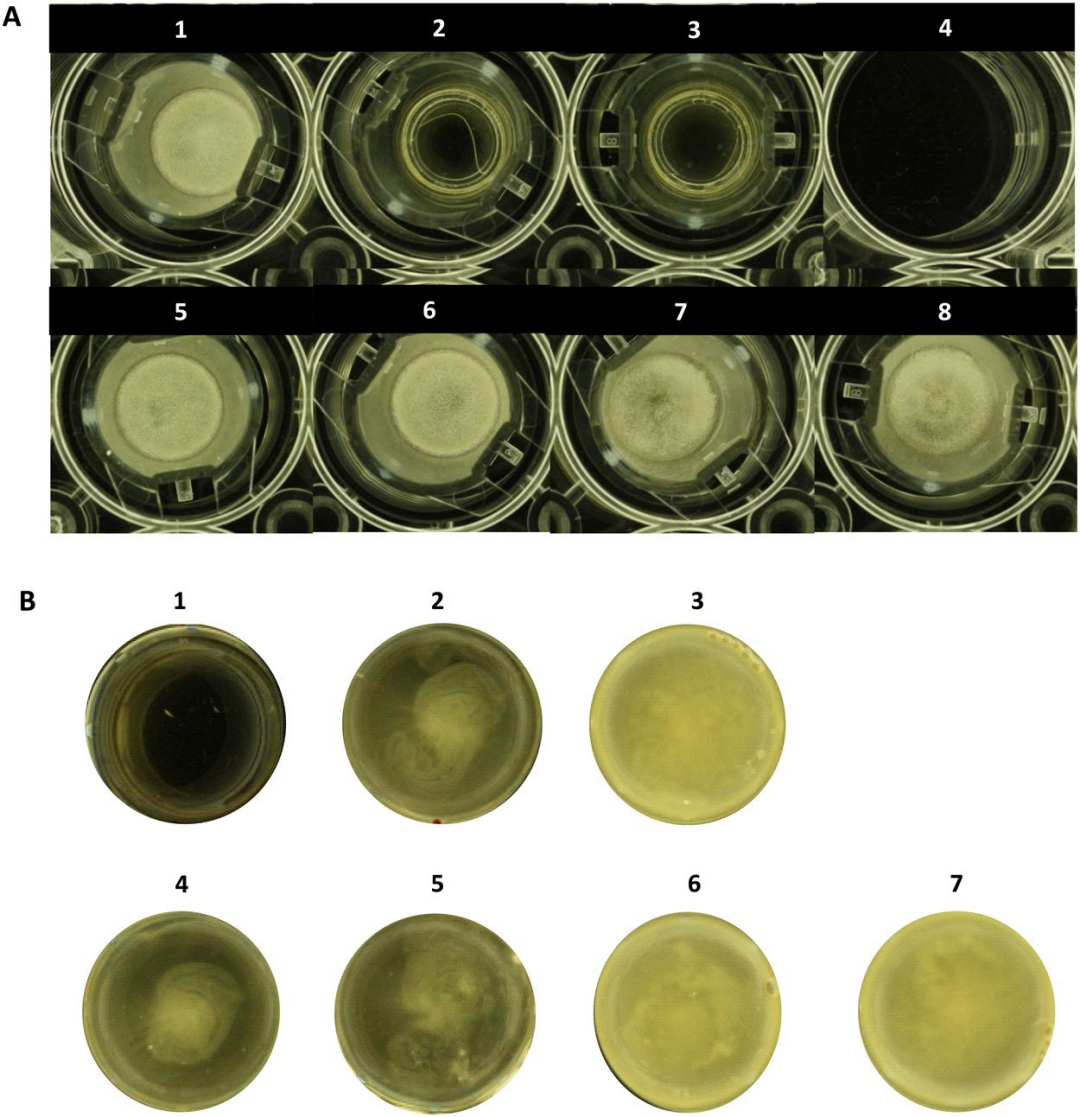
Physical interaction between *A. fumigatus* and *K. pneumoniae*. The bacteria and fungi were grown in Transwell plates providing physical separation, but permitting interaction by exchange of secreted molecules. (**A**) Photos of the inserts (upper compartments). 1. Growth of *A. fumigatus* alone; 2./3. No growth of the *K. pneumoniae* strains *K.p*. 13883 and *K.p*. 700603 which had been seeded solely in the lower compartments (see **B**); 4. Negative control: YPD; 5. *Aspergillus* growing in the upper compartment in the presence of *K.p*. 13883 in the lower compartment; 6. *Aspergillus* growing in the upper compartment in the presence of a co-culture of *A.f. vs K.p*. 13883 in the lower compartment; 7. *Aspergillus* growing in the upper compartment in the presence of *K.p*. 700603 in the lower compartment; 8. *Aspergillus* growing in the upper compartment in the presence of a co-culture of *A.f. vs K.p*. 700603 in the lower compartment. (**B**) Photos of the wells (lower compartments) reflecting *K. pneumoniae* grown alone or in combination with *A. fumigatus*. The fungus seeded in the upper compartment cannot transgress to the lower compartment and vice versa. The wells show presence of bacterial growth only, without any evidence for *A. fumigatus* growth. 1. *A. fumigatus* without fungal growth as the fungus was seeded in the upper compartment (see **A**); 2. Growth of *K.p*. 13883 alone in the lower compartment; 3. Growth of *K.p*. 700603 alone in the lower compartment; 4. *K.p*. 13883 growing in co-culture with *A.f*. in the lower compartment, without any fungus in the upper compartment; 5. *K.p*. 13883 growing in co-culture with *A.f*. in the lower compartment with fungal growth in the upper compartment (see **A**); 6. *K.p*. 700603 growing in co-culture with *A.f*. in the lower compartment, without any fungus in the upper compartment; 7. *K.p*. 700603 growing in co-culture with *A.f*. in the lower compartment with fungal growth in the upper compartment (see **A**).

Based on the documented antagonistic effect of *K. pneumoniae* strains against the development and growth of *Aspergillus* species, we have addressed the contact-dependence of the interaction. Transwell plates permitting the growth of bacteria and fungi in physically separated compartments but allowing exchange of diffusible molecules through a porous membrane were employed for the analysis. In the wells (lower compartments), *K. pneumoniae* strains were grown alone or in co-culture with *A. fumigatus*, whereas in the inserts with the porous membrane (upper compartments), *A. fumigatus* was grown alone. This strategy allowed us to investigate whether the inhibitory effect on *A. fumigatus* was mediated via direct contact with *K. pneumoniae* or by the secretion of molecules conferring independence from physical contact. The results have shown that *A. fumigatus* was able to grow in the upper compartments physically separated from *K. pneumoniae* growing in the lower compartments (Fig. 3A), indicating dependence on direct contact for the inhibitory effect.

The lower compartments, where bacteria were grown alone or in co-culture with *A. fumigatus*, are shown in Fig. 3B. The presence of *K. pneumoniae* alone or in co-culture in the lower compartment did not result in any inhibitory effect on *A. fumigatus* in the upper compartment.

To further assess the putative secretion of inhibitory molecules, we investigated the effect of the bacterial supernatant (SN) on *A. fumigatus* growth. Supernatants of *K. pneumoniae* grown alone or in co-culture in biofilm mode were obtained at 12 h and 24 h. Collection of supernatants at different time points was performed to determine if the inhibitory effect is growth phase-dependent, because the secretion of molecules might vary during the bacterial growth cycle. The supernatants were added to cultures of *A. fumigatus*. After 48 h of incubation, the results have shown that, regardless of the culture conditions or growth phase of the bacteria, various preparations of bacterial SN were unable to inhibit growth of any of the fungal *Aspergillus* species studied (Fig. S1). By contrast, in the presence of bacteria, either in exponential or stationary growth phase, the inhibitory effect on *Aspergillus* growth was observed (Fig. S2). By contrast, co-cultures with heat- or UV-killed *K. pneumoniae* did not show any reduction of fungal growth, demonstrating the requirement of live bacteria for the inhibitory effect (Fig. 5A).

### *Aspergillus* species remain viable upon interaction with *K. pneumoniae*

**Figure 4:**
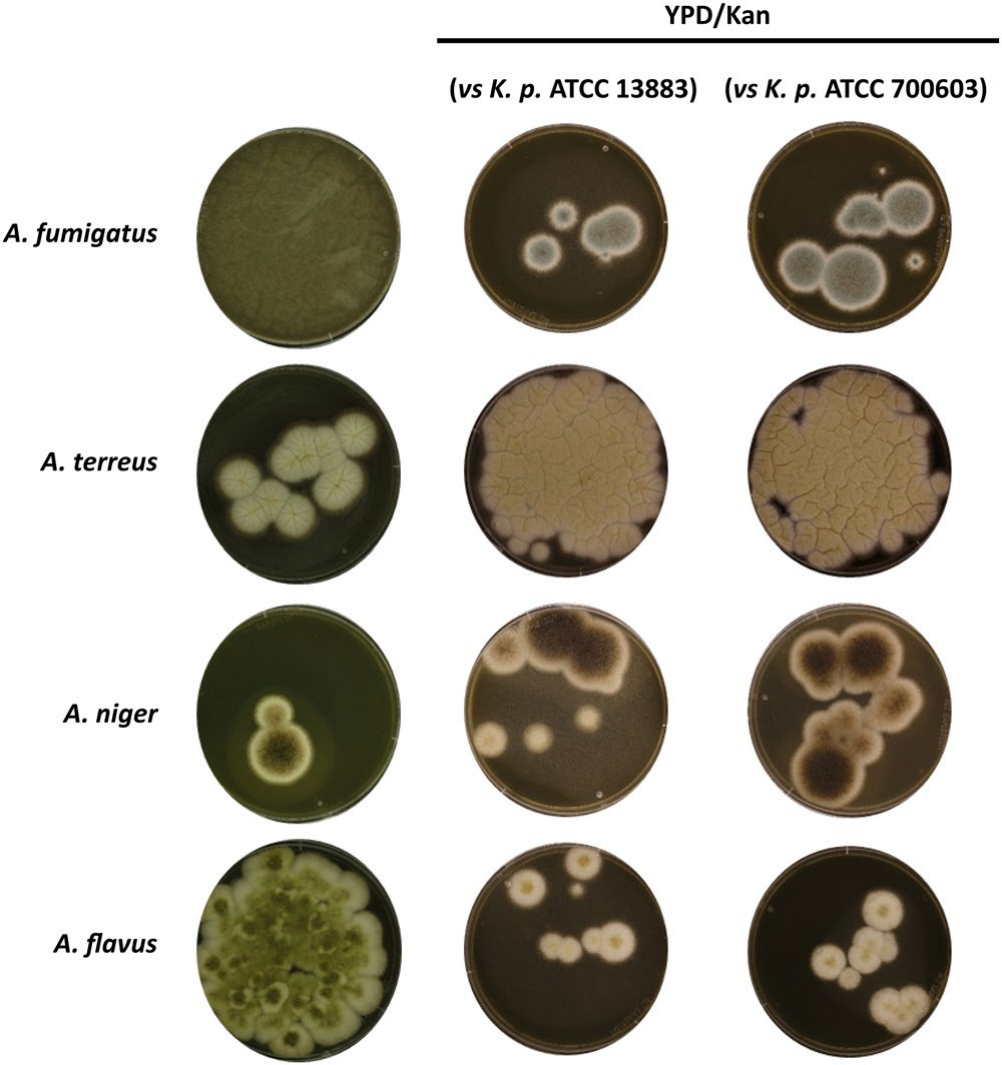
Viability of *Aspergillus* spp. upon interaction with *K. pneumoniae. Aspergillus* species grown alone and in co-culture with different *K. pneumoniae* strains for 24 h were aliquoted onto agar plates containing YPD (controls) and YPD plus the antibiotic kanamycin for selection of fungal growth. Images show fungal growth after incubation for 48 h.

**Figure 5:**
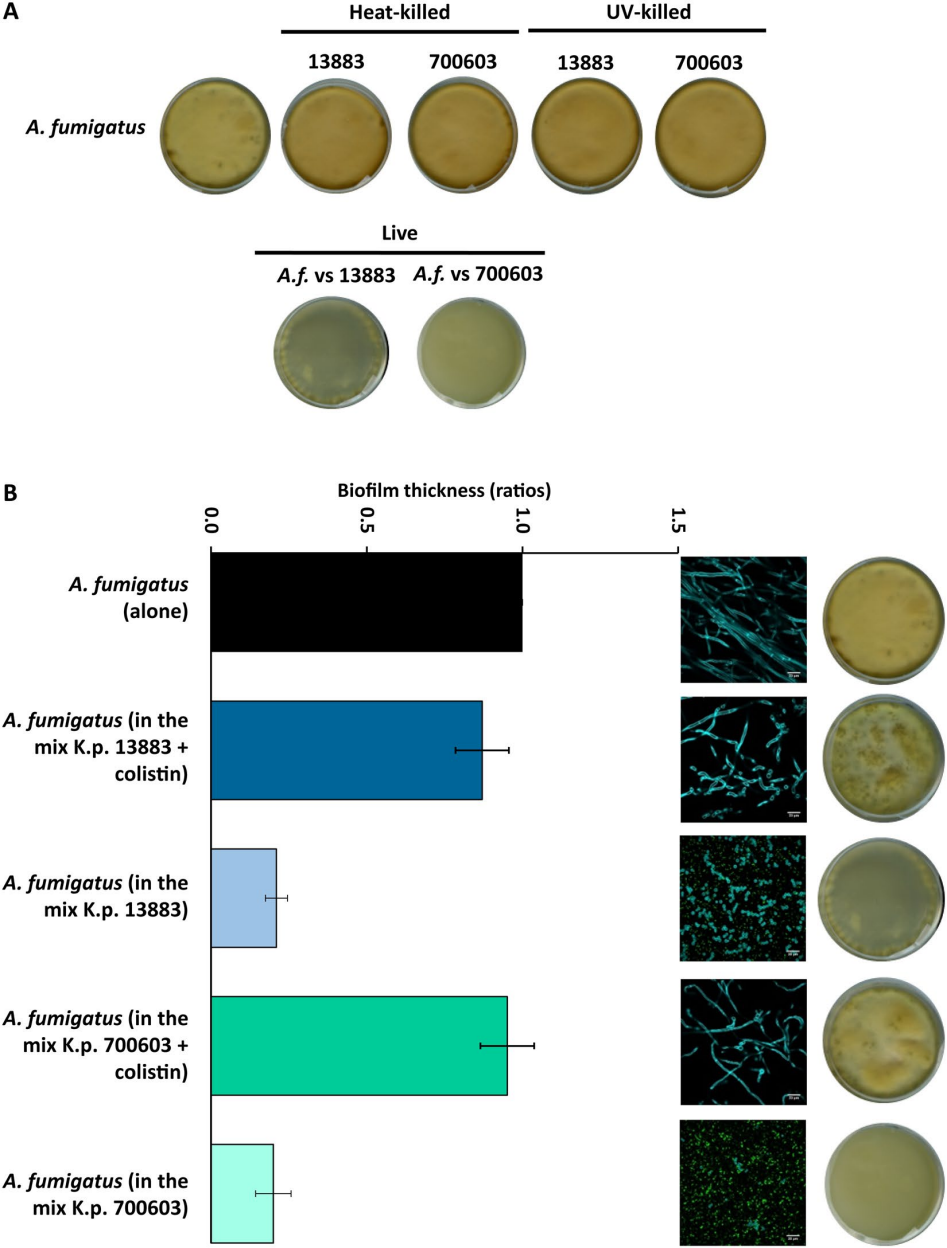
Growth recovery of *A. fumigatus* after exposure to heat-killed, UV-killed and antibiotic-treated *K. pneumoniae*. (**A**) Scanner photos of the bottom of tissue culture dishes after 48 h of culture revealing fungal growth recovery in the presence of heat- and UV-killed *K. pneumoniae* strains. Controls are shown for live *A. fumigatus vs K. pneumoniae* 13883 and 700603, revealing inhibition of fungal growth. (**B**) The plot shows biofilm thickness of *A. fumigatus* alone and after exposure to the *K. pneumoniae* strains tested, upon treatment with colistin, indicating fungal growth recovery in the presence of antibiotic-treated bacteria. Decreased biofilm thickness of *A. fumigatus* in the presence of non-inhibited *K. pneumoniae* 13883 or 700603 is also shown as a control experiment. Confocal images and dishes are representative of single and co-cultures upon incubation for 48 h. Scale bars are 20 µm. Data are presented as mean + SE of three independent experiments.

In order to assess whether the observed inhibitory effect of *K. pneumoniae* on the growth and development of various *Aspergillus* species is sustainable or reversible, the fungi were grown alone or in co-culture with *K. pneumoniae*. After 24 h growth in biofilm mode, aliquots of the single cultures and the co-cultures were streaked onto YPD agar plates containing the antibiotic kanamycin to prevent bacterial growth. The cultures were evaluated after 48 h of incubation, and confirmed efficient elimination of the bacteria. The co-cultures of *K. pneumoniae* strains with *Aspergillus* species revealed recovery of fungal growth upon exposure to kanamycin (Fig. 4), demonstrating that the fungi maintain their viability and ability to re-initiate growth once the inhibitory effect of the bacteria is eliminated. The same effect was observed when using other antibiotics including colistin or tetracycline which target different structures of the bacteria (Fig. S3). Confocal microscopy was used to assess quantitatively the growth recovery of *A. fumigatus* upon interaction with *K. pneumoniae* and subsequent treatment with an antibiotic. Co-cultures of *A. fumigatus* and *K. pneumoniae* were initiated at time zero, followed by antibiotic treatment with colistin at 6 h for elimination of the bacteria. After 24 h growth in co-culture or alone, measurements of the biofilm thickness of *A. fumigatus* revealed that, in the antibiotic-treated cultures, *A. fumigatus* biofilms were similar to *A. fumigatus* in single culture. The respective confocal microscopy images also showed hyphal development, contributing to the biofilm thickness. In addition, the Petri dishes also showed growth recovery of *A. fumigatus* in co-culture after antibiotic treatment, similar to cultures of the fungus growing alone (Fig. 5B).

### Growth inhibition of *Aspergillus* species by *K. pneumoniae* is not affected by nutrient shortage

Nutrient competition for the carbon source is a potential limiting factor for fungal growth during the interaction of *K. pneumoniae* with *Aspergillus* spp. To address this notion, the interaction between *A. fumigatus* and *K. pneumoniae* (ATCC strain 700603), YPD containing a standard glucose concentration of 2% was compared with YPD containing 4% glucose. The microorganisms were grown alone and in co-culture for 48 h in YPD containing the indicated glucose concentrations. No major differences in fungal growth were observed during the interaction with bacteria (Fig. S4), suggesting that the availability or lack of carbon was not a relevant factor for the antagonistic effect exerted by the bacteria on fungal growth.

### *K. pneumoniae* renders *A. fumigatus* sensitive to cell wall stress and induces upregulation of cell wall-related genes

**Figure 6:**
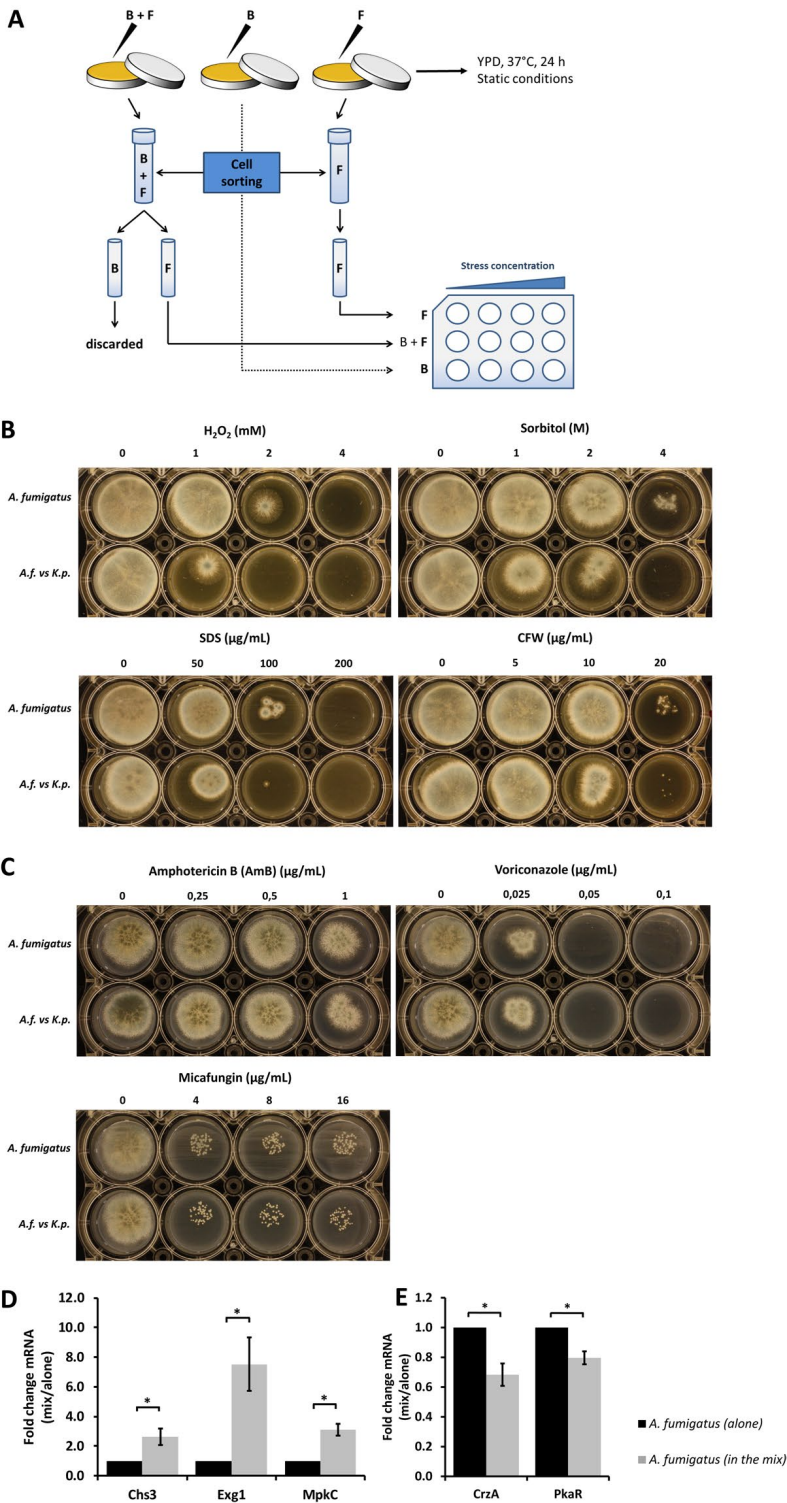
Induction of cell wall stress and upregulation of cell wall-related genes in *A. fumigatus* mediated by *K. pneumoniae*. (**A**) Schematic view of flow sorting of *A. fumigatus* and *K. pneumoniae* (ATCC 700603). Sorted cells were plated onto 12-well agar plates containing kanamycin (1000 µg/mL) and colistin (100 µg/mL) for selection of fungal growth and prevention of any residual bacterial contamination. Plates also contained different concentration ranges of cell wall stressors and antifungal drugs to facilitate the observation of the sensitivity of fungi previously grown alone or exposed to *K. pneumoniae*. (**B**) Sensitivity of *A. fumigatus* to oxidative (H_2_O_2_), osmotic (sorbitol, SDS) and cell wall (CFW) stresses, with and without previous exposure to *K. pneumoniae*. (**C**) Sensitivity of *A. fumigatus* to antifungal drugs (amphothericin B, voriconazole, micafungin) with and without previous exposure to *K. pneumoniae*. (**D**) Upregulation of *A. fumigatus* cell wall-related genes in the presence of *K. pneumoniae*. (**E**) Downregulation of hyphae-related genes of *A. fumigatus* in the presence of *K. pneumoniae* determined by quantification of mRNA expression of hyphae-related genes of *A. fumigatus* upon exposure to *K. pneumoniae* (ATCC 700603). Fungal spores were germinated for up to 12 h followed by addition of bacteria for a total of 24 h. Transcript levels of the *A. fumigatus* single culture were set to one, and the transcript levels in the co-culture were normalized to the single culture. Additionally, normalization was also performed against the reference gene *TUBA*. Data are presented as mean + SE of four independent experiments.

We investigated whether *K. pneumoniae* could induce cell wall stress in *A. fumigatus* and render the fungus more or less susceptible to stressors including antifungal drug treatment. After 24 h of growth in single culture or co-culture, *A. fumigatus* and *K. pneumoniae* were isolated by flow sorting. Equal numbers of sorted cells were seeded into 12-well agar plates containing a combination of the antibiotics kanamycin and colistin to prevent bacterial growth. Moreover, the plates contained different concentrations of oxidative (H_2_O_2_), osmotic (sorbitol, sodium dodecyl sulfate (SDS)) and cell wall stressors (Calcofluor white (CFW)) as well as antifungal drugs (Fig. 6A). The sensitivity to these stresses was compared between *A. fumigatus* grown alone or upon exposure to *K. pneumoniae*, and the latter constellation revealed at least two-fold greater sensitivity (Fig. 6B). Additionally, three different classes of antifungal drugs including Amphotericin B, Voriconazole and Micafungin were tested to assess the sensitivity of *A. fumigatus* grown under the conditions indicated above. However, no differential sensitivity has been observed (Fig. 6C).

To address the genetic basis of stress response of *A. fumigatus*, expression analysis of cell wall-related genes was performed at 24 hours after adding *K. pneumoniae* to *A. fumigatus* cultures pre-germinated for 12 h. Transcript levels of three genes were quantified by qPCR including *CHS3*, a regulator of chitin synthase expression, *EXG1*, a conveyer of glucan β-glucosidase activity, *and MPKC*, a putative mitogen-activated protein kinase (MAPK) involved in oxidative stress response. Normalization was performed against the reference gene *TUBA*^107–109^ and *A. fumigatus* grown alone. Significant upregulation of the *A. fumigatus* genes *CHS3, EXG1 and MPKC* was documented upon exposure to *K. pneumoniae* (Fig. 6D). Conversely, significant downregulation of genes associated with hyphal development such as *CRZA* and *PKAR* was identified (Fig. 6E). *CRZA* is a transcription factor in the calcineurin pathway, regulating conidial germination, hyphal growth and virulence^110–113^, and *PKAR* is a protein kinase in the cyclic adenosine monophosphate (cAMP) pathway also regulating hyphal formation^114,115^.

## Discussion

We have demonstrated that the interaction between different strains of *K. pneumoniae* and various *Aspergillus* species *in vitro* mediates inhibition of fungal spore germination and hyphal growth. In addition, *K. pneumoniae* was also able to impair biofilm formation of *Aspergillus* species. This effect was shown to require the presence of live, actively growing bacteria and to be dependent on physical contact between the microorganisms. The inhibitory effect was reversible, as revealed by the resumption of fungal growth upon elimination of the bacteria by antibiotic treatment. During the interaction with *K. pneumoniae, Aspergillus* adopted a dormant or standby mode, during which the genes regulating hyphal development were downregulated, conferring low energy supply requirement for survival. This notion is in accordance with the observation that the effects of the interaction were apparently independent of more or less abundant carbon source, suggesting that competition for nutrients may not be a major factor in the inhibition of fungal growth.

Moreover, our data suggest that *K. pneumoniae* confers stress to the fungal cells resulting in the upregulation of protective mechanisms which involve remodelling and reinforcement of the fungal cell wall. This effect was demonstrated by increased sensitivity to stress induced by agents conferring osmotic, oxidative or cell wall-targeted challenges. In response to the stress effects, *Aspergillus* showed elevated expression of the *CHS3, EXG1 and MPKC* genes, which are involved in cell wall remodelling and oxidative stress response. This observation is in line with earlier studies indicating that, upon exposure to stress, fungi initiate a response based on activating cell wall salvage pathways that compensate for cell wall damage as a rescue mechanism^96–100^ including, for example, an increase in transcript abundance of *MPKC* in response to oxidative stress^102–104,116–118^. Since the formation of biofilms can per se lead to resistance against antimicrobial treatment owing to the decreased physical accessibility of the pathogens by drugs^89,119–121^, additional effects of microbial interactions within mixed biofilms affecting the protective mechanisms of individual pathogens may further enhance the resistance to therapy. The understanding of processes occurring during bacterial-fungal interactions (BFI) is therefore essential for the development of appropriate diagnostic and therapeutic approaches. The inhibitory or stimulating effect of BFI mediated by secreted molecules has been demonstrated for different constellations involving molecular cross-talk via production of quorum sensing molecules^12–16,18–20,25,26,122^. Earlier observations of BFI involving *K. pneumoniae* and *C. albicans* revealed a similar antagonistic effect mediated by the bacteria, but the mechanisms of the interaction have remained unclear^123^. By contrast, an example for synergistic effects is a report on the interaction between *Klebsiella aerogenes* and *Cryptococcus neoformans*, which revealed the induction of melanin production in the fungus mediated by bacterial secretion of dopamine, leading to enhanced protection of *Cryptococcus* from macrophages^12^. *K. pneumoniae* was also found to increase spore germination and hyphal growth of *Glomus deserticola*, a vesicular-arbuscular mycorrhizal fungus, and the authors suggested that *K. pneumoniae* may produce a diffusible compound resulting in hyphal extension^124^.

It is important to point out that the observations of BFI *in vitro* may not necessarily be reflected by identical implications *in vivo*, as demonstrated for the interaction between *C. albicans* and *P. aeruginosa* which were shown to display inhibitory effects *in vitro*^13,32,125,126^ but synergistic effects in mouse and zebrafish *in vivo* models^127,128^. Co-infections affecting the lungs of patients with cystic fibrosis resulted in worse clinical outcome in comparison to infections with each of the pathogens individually^14,21,35–37^.

Although this observation may not necessarily indicate discrepant *in vitro* and *in vivo* effects of the BFI, it highlights the need to verify observations made *in vitro* by exploiting models reflecting the complexity encountered in the human host organism. Such analyses may involve the use of organoids generated from primary biopsy materials or animal models. We are currently generating intestinal organoids derived from colon and jejunum/ileum biopsies from patients undergoing diagnostic endoscopy in order to facilitate studies of BFI in a system mimicking the human bowel. Moreover, we are planning to study the interactions in a zebrafish model which permits analyses in the presence of an immune system closely resembling the situation in the human host^129,130^. Once confirmed in additional model systems, the observations presented may have important clinical implications. If, in the presence of co-localized infections by *K. pneumoniae* and various *Aspergillus* species, treatment with antibiotics could indeed unleash fungal growth, it may lead to rapid expansion of *Aspergillus* requiring timely therapeutic intervention to prevent severe and potentially life-threatening disease in immunocompromised hosts. The identification of metabolites, such as specific quorum sensing molecules released during BFI, could be exploited for clinical diagnosis of these co-infections, and provide a basis for appropriate treatment measures. Proteomic, metabolomic and genetic analyses of the interaction between *K. pneumoniae* and *Aspergilli* are currently ongoing, and will expectedly reveal candidate molecules for future diagnostic exploitation, with the ultimate aim to improve the control and outcome of polymicrobial infections, particularly in the immunocompromised patient setting.

## Materials and Methods

### Strains and growth conditions

*Aspergillus* and *Klebsiella pneumoniae* strains used in this study are listed in Table 1. Two different strains of *K. pneumoniae* were used, a low-biofilm (ATCC 13883) and a high-biofilm (ATCC 700603) forming strain^75^. *Aspergillus* strains were maintained in Malt Extract agar (MEA, Sigma) plates for 3–4 days at 37 °C. Spores were collected into 1x PBS + 0.1% Tween 20, following filtration by using a 40 μm cell strainer. Spores were stored at 4 °C for up to one week for subsequent use in the experiments. *K. pneumoniae* strains were maintained in Luria-Bertani (LB) agar plates and grown overnight in liquid LB medium at 37 °C with agitation at 180 rpm. Equal cell numbers of *Aspergillus* spp. and *K. pneumoniae* (10^6^ cells/mL) were used in co-culture experiments. For qualitative assessment of bacterial-fungal interaction, *Aspergillus* spp. and *K. pneumoniae* were grown alone and in co-culture in sterile-filtered yeast extract peptone dextrose (YPD) medium (Formedium, Norfolk, UK) at 37 °C. The microorganisms were grown as biofilms, with static incubation in 35 × 10 mm tissue culture dishes (CytoOne, Starlab GmbH, Ahrensburg, Germany). For co-culture experiments, fungi and bacteria were either mixed at time zero or *K. pneumoniae* strains were added after pre-germination of *Aspergillus* spores, permitting the formation of hyphae for up to 12 hours (h). For analysis by confocal microscopy, the same growth procedure was performed using IBIDI plates (µ-dish, 35 mm high, ibiTreat, 35 mm). Growth phase tests of *K. pneumoniae* included overnight growth for 16 h (stationary phase). For exponential phase, *K. pneumoniae* was grown overnight, followed by growth in fresh medium for 3 additional h.

**Table 1:** Microbial strains and primers used for PCR analysis.

		Designation
**Strains**	**Species**	**Code**
	*A. fumigatus*	ATCC 204305
	*A. terreus*	ATCC 1012
	*A. niger*	DSM 1959
	*A. flavus*	CM 5095
	*K. pneumoniae*	ATCC 13883
		ATCC 700603
**Primers**	***Aspergillus***	**Sequence (5′ to 3′)**
	28S_Fw	GTTGTTTGGGAATGCAGCTCTA
	28S_Rv	TCTCCGGCCAGTATTTAGCTTT
	Chs3_Fw	TAGCCAGAACAACTCCTCCC
	Chs3_Rv	TGAGTGCGACCTTAGAATTACGA
	Exg1_Fw	AGATTACTACAACCAGATTGCGG
	Exg1_Rv	GTATCCATGACCACATCCTCAC
	MpkC_Fw	CCACCTCATCACAAACATCCT
	MpkC_Rv	GGCATCGAAATCAGTATCTTTGG
	CrzA_Fw	GAGAACTTCACCTTGTCCGAG
	CrzA_Rv	GGCATCATTTCCTGTCCCTG
	PkaR_Fw	CATCCGAAGACCGAAGAACAG
	PkaR_Rv	CCAAAGCGTCAAGTACAGTCC
	TubA_Fw	GGTAACCAAATCGGTGCTGCTTTC
	TubA_Rv^133^	ACCCTCAGTGTAGTGACCCTTGGC
	***Klebsiella***	**Sequence (5′ to 3′)**
	16S_Fw	CCAGCAGCCGCGGTAA
	16S_Rv	TTACGCCCAGTAATTCCGATTAA

ATCC (American type culture collection), DSM (Deutsche sammlung von mikroorganismen und zellkulturen), CM (Centro nacional de microbiologia), Fw (forward), Rv (reverse). With the exception of primers for TubA^133^, all primers employed were specifically designed for the present study.

### Primer design and qPCR

Genomic sequences of *Aspergillus* spp. and *K. pneumoniae* were retrieved from the NCBI database (https://www.ncbi.nlm.nih.gov/), and species-specific primers were designed for amplification of the conserved 28 S and 16 S rRNA sequences for fungi and bacteria, respectively. For gene expression analysis by qPCR, target sequences of *Aspergillus* spp. and *K. pneumoniae* were retrieved from the *Aspergillus* genome database (http://www.aspgd.org/) and the Kyoto encyclopedia of genes and genomes (KEGG) (http://www.genome.jp/kegg/), respectively. Primer design was performed by using the software PerlPrimer (version 1.1.21) (Open-source PCR primer design, Parkville, Australia)^131^. Fasta files were uploaded and the features selected included annealing temperatures of 60–62 °C and amplicon sizes of 100–150 bases. Primers used in this study are listed in Table 1. Primers were pre-tested for correct amplification prior to use in qPCR analysis. The thermocycler used for qPCR reactions was the Realplex Mastercycler epgradient S, Eppendorf (Hamburg, Germany).

### Transformation of *K. pneumoniae* with GFP

Strains of *K. pneumoniae* ATCC 13883 and ATCC 700603 were transformed with the plasmid pUA-PrpsM-gfp, ORI: SC101, with GFP and kanamycin markers^132^. Briefly, competent cells were prepared with CaCl_2_, following transformation by electroporation (2,5 KV for 0,2 cm gap cuvette or 1,75 KV for 0,1 cm gap cuvette, 200 Ω, 25 μF, time constant ≥4 msec), using 1 µL plasmidic DNA. Cells were then recovered in pre-warmed LB medium and positive transformants selected on kanamycin agar plates. Uptake of the plasmid was confirmed by flow cytometry and fluorescence microscopy.

### Imaging by confocal microscopy and biofilm thickness measurements

*Aspergillus* and *K. pneumoniae* cells were visualized under a confocal laser scanning microscope (CLSM) using IBIDI plates (µ-dish, 35 mm high, ibiTreat, 35 mm). Prior to imaging, cells were fixed with 4% paraformaldehyde for 30 min, followed by calcofluor white (CFW) staining (10 µg/mL) of single and co-cultures of *Aspergillus* for 20 min in the dark, at room temperature. *K. pneumoniae* strains were GFP-tagged as described above. The fluorescence channels for DAPI and EGFP were applied to image *Aspergillus* and *K. pneumoniae* cells, respectively. For biofilm thickness (µm) measurements of single and co-cultures, the Z-stack function was selected. Images were analyzed using the Fiji software (Open source Java image processing, NIH image).

### Transwell plates assay

The effect of physical contact on the interaction between *Aspergillus* and *K. pneumoniae* was assessed in Transwell® plates (6.5 mm diameter inserts with 0.4 µm Pore Polyester Membrane, tissue culture-treated, Corning, Costar). These plates contain an upper compartment or insert which is separated from the lower compartments or wells. Briefly, YPD medium was pre-warmed and 600 µL were placed in the wells, while 100 µL were placed into the inserts. *A. fumigatus* was seeded into the inserts and the wells contained either *K. pneumoniae* alone or in co-culture with *A. fumigatus*. Imaging of the plates was performed after 24 h incubation at 37 °C with a camera (Canon Macro Lens EF-S 60 mm). Potential cross-over of microorganisms between the upper and lower compartments was excluded by microscopy-based control.

### Supernatant assay

Supernatant (SN) of *K. pneumoniae* growing alone and in co-culture with *Aspergillus* spp. was obtained after 12 and 24 h growth in biofilm mode in 35 × 10 mm tissue culture dishes (CytoOne, Starlab GmbH, Ahrensburg, Germany). After growth, cells and supernatants were collected into Eppendorf tubes and spun down 2x at maximum speed (25 000 g) at 4 °C (centrifuge 5417 R, Eppendorf, Hamburg, Germany). The SNs corresponding to each growth condition were pooled into 50 mL Falcon tubes, followed by filtration with vacuum filter units (Millipore Express PLUS (PES) 0.22 µm membrane). The freshly collected SN was immediatelly used for growth of *Aspergillus* spp. Different concentrations of YPD medium were used, including 0.5 volumes SN and 0.5 volumes of 2x YPD or 1x YPD or H_2_O. These conditions were intended to mimic exhaustion of nutrients, availability of nutrients and non-exhausted SN, respectively. Imaging of the plates was performed after 48 h incubation. As a control, to check for cell-free supernatants, an aliquot (100 µL) of each SN was distributed on LB agar plates and incubated at 37 °C.

### Testing for recovery of fungal viability

*Aspergillus* spp. and *K. pneumoniae* were grown alone and in co-culture in sterile-filtered YPD medium (Formedium, Norfolk, UK) at 37 °C. These were grown as biofilms with static incubation in 35 × 10 mm tissue culture dishes (CytoOne, Starlab GmbH, Ahrensburg, Germany). Co-cultures of fungi and bacteria were mixed together at time zero and incubated for 24 h. An aliquot of these cultures (100 µL) was then streaked on YPD agar plates containing the antibiotic kanamycin 1000 µg/mL to select for fungal growth. Imaging was performed (Canon Macro Lens EF-S 60 mm) after 48 h incubation at 37 °C. As a negative control, to check for absence of bacterial growth, *K. pneumoniae* strains were streaked alone onto YPD/Kanamycin agar plates. Positive controls were implemented by growing *Aspergillus* spp. alone in YPD/Kanamycin agar plates to check for unaffected fungal growth in the presence of this antibiotic.

### Heat-, UV-killed and antibiotic-treated bacteria

Heat-killing (HK) of *K*. *pneumoniae* cells was performed in a Thermomixer comfort (Eppendorf) at 95 °C for 30 min in PBS, whereas UV-killing (UVK) was performed in a Stratalinker for 3 cycles at 9999 × 100 µjoules with plate shaking between the cycles. Following HK or UVK, *K*. *pneumoniae* cells were added to *Aspergillus* spp in co-culture in 35 × 10 mm tissue culture dishes (CytoOne, Starlab GmbH, Ahrensburg, Germany) as described above (Strains and growth conditions). Imaging was performed after 48 h incubation. As a control, to check for efficiency of killing, an aliquot (100 µL) of HK and UVK cells was streaked onto LB agar plates and incubated at 37 °C.

Treatment of *K*. *pneumoniae* with the antibiotics colistin, kanamycin or tetracycline was performed at the concentrations of 100, 2000 and 10 µg/mL, respectively. *Aspergillus* spp. and *K. pneumoniae* were grown alone and in co-culture in YPD medium at 37 °C in 35 × 10 mm tissue culture dishes (CytoOne, Starlab GmbH, Ahrensburg, Germany). Co-cultures of fungi and bacteria were mixed together at time zero, and antibiotics were added to the culture at 6 h. Imaging was performed after 48 h incubation.

### Cell sorting of *A. fumigatus* and cell wall stress assay

Sorting of *A. fumigatus* cells growing alone or in co-culture with *K. pneumoniae* was performed after 24 h growth in 35 × 10 mm tissue culture dishes (CytoOne, Starlab GmbH, Ahrensburg, Germany). Following growth, cultures were collected into 15 mL Falcon tubes, spun down for 5 min at 4.400 g and SN was discarded. A volume of 2 mL 1x PBS was added and samples were vortexed vigorously. An aliquot (1 mL) was added into a FACS tube through a membrane on the lid. These lids were removed and CFW was added to the cells at a final concentration of 10 µg/mL. Samples were then processed using a FACSaria cell sorter (BD Biosciences) by sorting the cells based on CFW fluorescence (BV421 detector). A total of 20 000 cells were sorted into 1 mL 1x PBS. From these, a volume of 10 µL was spotted onto 12-well agar plates.

To perform the cell wall stress assay, 12-well agar plates were prepared using YPD agar containing the antibiotics colistin at 100 µg/mL and kanamycin at 1000 µg/mL to preclude any possibility of bacterial growth. In addition, each plate was complemented with different concentrations of chemical cell wall stressors and antifungals. The cell wall stresses included: H_2_O_2_ 1 mM, 2 mM and 4 mM; sorbitol 1 M, 2 M and 4 M; sodium dodecyl sulfate (SDS) 50 µg/mL, 100 µg/mL and 200 µg/mL; CFW 5 µg/mL, 10 µg/mL and 20 µg/mL. The antifungal drugs and their corresponding concentrations included amphotericin B (AmB) 0.25 µg/mL, 0.5 µg/mL and 1 µg/mL; voriconazole 0.025 µg/mL, 0.05 µg/mL and 0.1 µg/mL; micafungin 4 µg/mL, 8 µg/mL and 16 µg/mL. Plates were incubated at 37 °C and imaged (Canon Macro Lens EF-S 60 mm) after 2 days. *K. pneumoniae* growing alone was also spotted onto these plates as a control for complete growth inhibition of bacterial cells.

### Statistics

The significance of differences between *Aspergillus-K. pneumoniae* co-cultures and single cultures was determined by using the T-test with one-tailed distribution for paired samples (Excel software). *P* values < 0.05 were considered significant. The calculations were based on at least three independent biological replicates.

## Electronic supplementary material

Supplementary data
