# Supplementary material for: Klebsiella pneumoniae prevents spore germination and hyphal development of Aspergillus species
Source: Sci Rep. 2019 Jan 18;9:218. doi: 10.1038/s41598-018-36524-8 (PMC6338788; doi:10.1038/s41598-018-36524-8)
Supplement: Supplementary file 1 — Supplementary data [file 41598_2018_36524_MOESM1_ESM.docx]

***Klebsiella pneumoniae* prevents spore germination and hyphal development of *Aspergillus* species**

Nogueira M.F.^1,2,3^, Pereira L.^1,2,3^, Jenull S. ^3^, Kuchler K.^3^, Lion T.^1,2,4*^


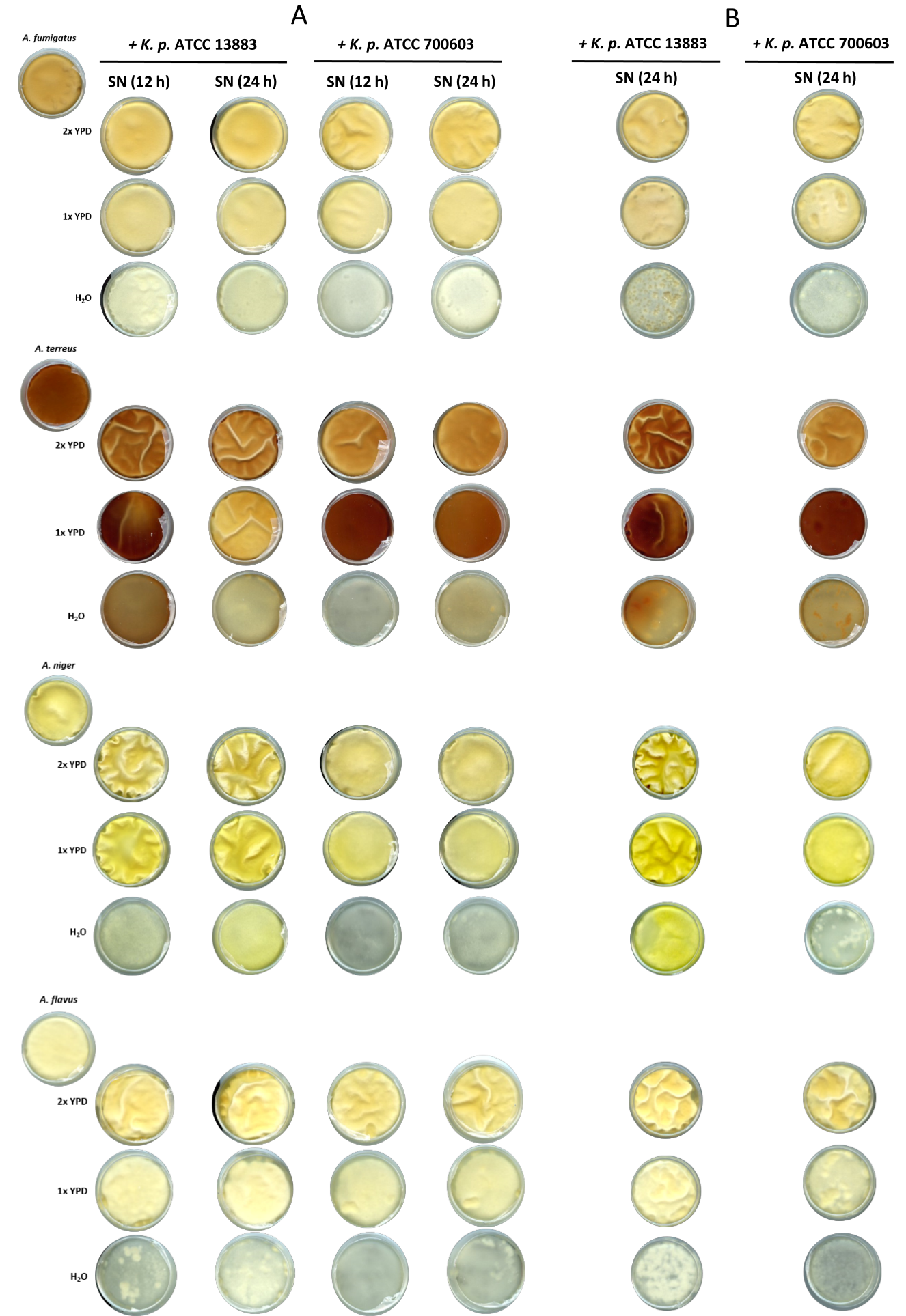


**Figure S1. Effect of *K. pneumoniae* supernatant on *Aspergillus* growth.** Supernatants from *K. pneumoniae* grown alone or mixed with *Aspergillus* spp. (biofilm mode) were added to 2x YPD, 1x YPD and H_2_O at a 1:1 ratio to grow *Aspergillus* spp. **A)** Supernatant from *K. pneumoniae* grown alone for 12 and 24 h, **B)** Supernatant from *K. pneumoniae* grown together with different *Aspergillus* spp. for 24 h. Imaging was performed at 48 h of incubation.


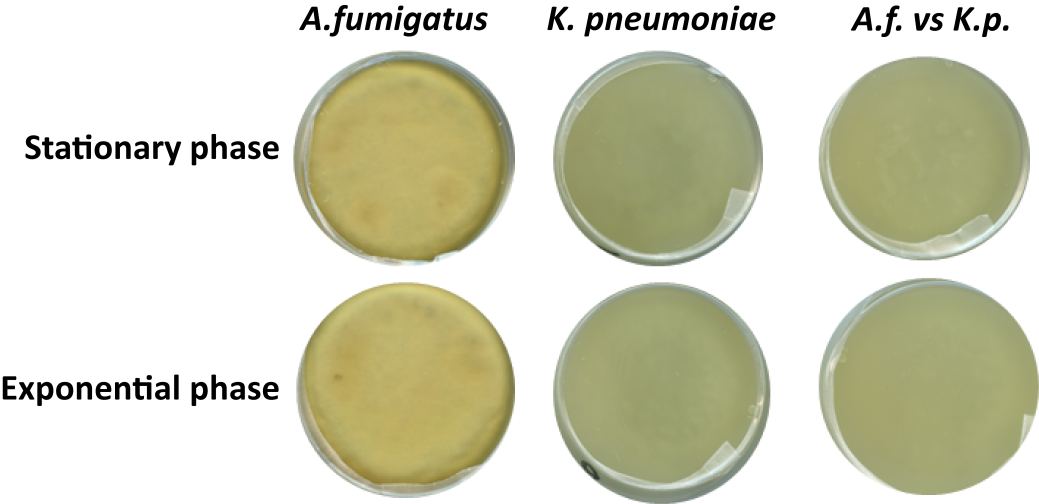


**Figure S2. Influence of *K. pneumoniae* growth phase on *A. fumigatus* growth.** *A. fumigatus* and *K. pneumoniae* (ATCC 700603) were grown in YPD, at 37°C in 35x10 mm tissue culture dishes (CytoOne) alone and in co-culture. Stationary phase: *K. pneumoniae* grown overnight for 16 h; Exponential growth: overnight cultures re-grown to exponential phase (+ 3 h in fresh medium), followed by co-culture with *A. fumigatus.* The scanner photos from the bottom of the dishes shown were taken after 48 h of incubation.


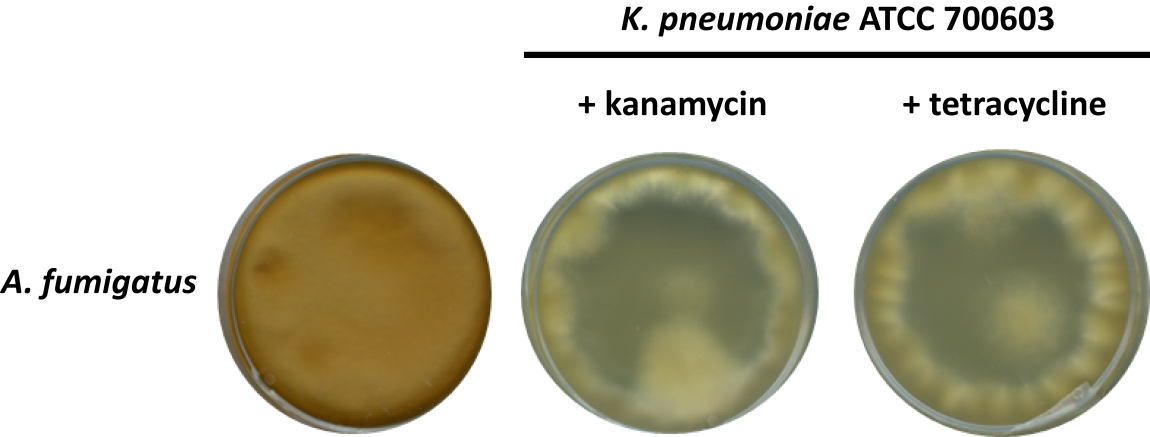


**Figure S3. *A. fumigatus* growth recovery after suppression of *K. pneumoniae* with the antibiotics kanamycin and tetracycline.** *A. fumigatus* and *K. pneumoniae* (ATCC 700603) were grown in YPD, at 37°C in 35x10 mm tissue culture dishes (CytoOne), alone and in co-culture. At 6 h, cultures were treated with the antibiotics kanamycin (2000 µg/mL) or tetracycline (10 µg/mL). The scanner photos from the bottom of the dishes shown were taken after 48 h of incubation.


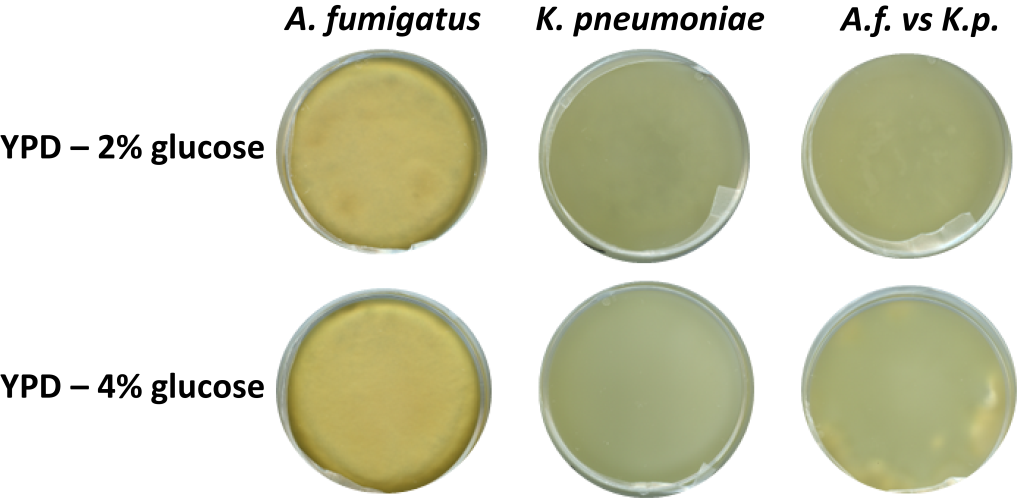


**Figure S4. Influence of additional glucose on *A. fumigatus* growth upon interaction with *K. pneumoniae.*** *A. fumigatus* and *K. pneumoniae* (ATCC 700603) were grown in YPD, at 37°C in 35x10 mm tissue culture dishes (CytoOne), alone and in co-culture. YPD medium was supplemented with 2% or 4% glucose. The scanner photos from the bottom of the dishes shown were taken after 48 h of incubation.
